# 5-Cyclo­hexyl-2-(4-fluoro­phen­yl)-3-methyl­sulfinyl-1-benzofuran

**DOI:** 10.1107/S1600536811002297

**Published:** 2011-01-22

**Authors:** Hong Dae Choi, Pil Ja Seo, Byeng Wha Son, Uk Lee

**Affiliations:** a Department of Chemistry, Dongeui University, San 24 Kaya-dong Busanjin-gu, Busan 614-714, Republic of Korea; b Department of Chemistry, Pukyong National University, 599-1 Daeyeon 3-dong, Nam-gu, Busan 608-737, Republic of Korea

## Abstract

In the title compound, C_21_H_21_FO_2_S, the cyclo­hexyl ring adopts a classic chair conformation. The 4-fluoro­phenyl ring makes a dihedral angle of 31.05 (6)° with the mean plane of the benzofuran fragment. In the crystal, mol­ecules are linked through weak inter­molecular C—H⋯O and C—H⋯π inter­actions.

## Related literature

For the biological activity of benzofuran compounds, see: Aslam *et al.* (2006 ▶); Galal *et al.* (2009 ▶); Khan *et al.* (2005 ▶). For natural products with benzofuran rings, see: Akgul & Anil (2003 ▶); Soekamto *et al.* (2003 ▶). For our previous structural studies of related 2-(4-fluoro­phen­yl)-3-methyl­sulfinyl-1-benzo­furan derivatives, see: Choi *et al.* (2009 ▶, 2010**a* ▶,b* ▶).
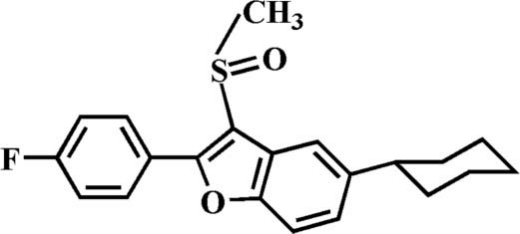


## Experimental

### 

#### Crystal data

C_21_H_21_FO_2_S
*M* *_r_* = 356.44
Orthorhombic, 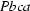

*a* = 16.4070 (6) Å
*b* = 11.3751 (4) Å
*c* = 18.7490 (7) Å
*V* = 3499.1 (2) Å^3^
*Z* = 8
Mo *K*α radiation
μ = 0.21 mm^−1^
*T* = 173 K
0.26 × 0.17 × 0.14 mm

#### Data collection

Bruker SMART APEXII CCD diffractometer
Absorption correction: multi-scan (*SADABS*; Bruker, 2009 ▶) *T*_min_ = 0.949, *T*_max_ = 0.972
17170 measured reflections
4007 independent reflections
3190 reflections with *I* > 2σ(*I*)
*R*_int_ = 0.041

#### Refinement

*R*[*F* ^2^ > 2σ(*F* ^2^)] = 0.040
*wR*(*F* ^2^) = 0.112
*S* = 0.93
4007 reflections
227 parameters
H-atom parameters constrained
Δρ_max_ = 0.32 e Å^−3^
Δρ_min_ = −0.44 e Å^−3^

### 

Data collection: *APEX2* (Bruker, 2009 ▶); cell refinement: *SAINT* (Bruker, 2009 ▶); data reduction: *SAINT*; program(s) used to solve structure: *SHELXS97* (Sheldrick, 2008 ▶); program(s) used to refine structure: *SHELXL97* (Sheldrick, 2008 ▶); molecular graphics: *ORTEP-3* (Farrugia, 1997 ▶) and *DIAMOND* (Brandenburg, 1998 ▶); software used to prepare material for publication: *SHELXL97*.

## Supplementary Material

Crystal structure: contains datablocks global, I. DOI: 10.1107/S1600536811002297/fl2333sup1.cif

Structure factors: contains datablocks I. DOI: 10.1107/S1600536811002297/fl2333Isup2.hkl

Additional supplementary materials:  crystallographic information; 3D view; checkCIF report

## Figures and Tables

**Table 1:** Hydrogen-bond geometry (Å, °) *Cg*1 and *Cg*2 are the centroids of the C1/C2/C7/O1/C8 furan ring and C9–C14 4-fluoro­phenyl ring.

*D*—H⋯*A*	*D*—H	H⋯*A*	*D*⋯*A*	*D*—H⋯*A*
C21—H21*B*⋯O2^i^	0.98	2.51	3.468 (2)	166
C13—H13⋯*Cg*1^i^	0.95	2.55	3.427 (2)	153
C20—H20*A*⋯*Cg*2^ii^	0.99	2.73	3.537 (2)	140

Symmetry codes: (i) 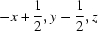; (ii) 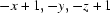.

**Fig. 1.**
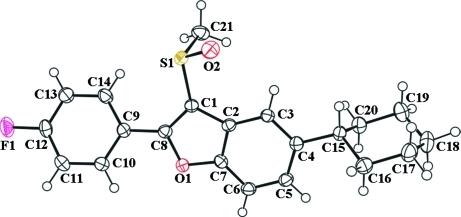
The molecular structure of the title compound with the atom numbering scheme. Displacement ellipsoids are drawn at the 50% probability level. H atoms are presented as a small spheres of arbitrary radius.

**Fig. 2.**
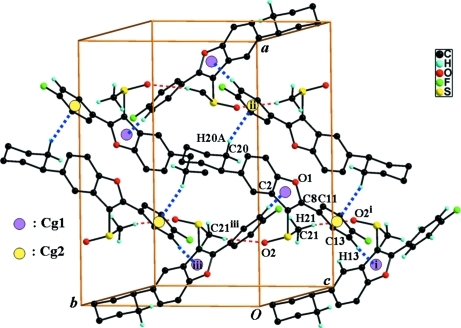
A view of the C—H···O and C—H···π interactions (dotted lines) in the crystal structure of the title compound. [Symmetry codes: (i) - x + 1/2, y - 1/2, z; (ii) - x + 1, - y , - z + 1 ; (iii) - x +1/2, y + 1/2, z.]

## supplementary crystallographic information

### Comment

Many compounds involving a benzofuran ring system have aroused much attention owing to their pharmacological properties such as antifungal, antimicrobial, antitumor and antiviral activities (Aslam *et al.*, 2006; Galal *et al.*, 2009; Khan *et al.*, 2005). These compounds occur in a wide range of natural products (Akgul & Anil, 2003; Soekamto *et al.*, 2003). As part of our ongoing program of the substituent effect on the solid state structures of 2-(4-fluorophenyl)-3-methylsulfinyl-1-benzofuran analogues (Choi *et al.*, 2009, 2010**a*,b*), we report herein on the crystal structure of the title compound.

In the title molecule (Fig. 1), the benzofuran unit is essentially planar, with a mean deviation of 0.017 (1) Å from the least-squares plane defined by the nine constituent atoms. The cyclohexyl ring is in the chair form. The 4-fluorophenyl ring makes a dihedral angle of 31.05 (6)° with the mean plane of the benzofuran ring. The crystal packing (Fig. 2) is stabilized by a weak intermolecular C–H···O interaction between a methyl H atom and the oxygen of the S═O unit (Table 1; C21—H21B···O2^i^). The crystal packing (Fig. 2) is further stabilized by intermolecular C–H···π interactions; the first one between a 4-fluorophenyl H atom and the furan ring (Table 1; C13—H13···Cg1^i^, Cg1 is the centroid of the C1/C2/C7/O1/C8 furan ring), and the second one between a cyclohexyl H atom and the 4-fluorophenyl ring (Table 1; C20—H20A···Cg2^ii^, Cg2 is the centriod of the C9-C14 4-fluorophenyl ring).

### Experimental

77% 3-chloroperoxybenzoic acid (202 mg, 0.9 mmol) was added in small portions to a stirred solution of 5-cyclohexyl-2-(4-fluorophenyl)-3-methylsulfanyl-1-benzofuran (306 mg, 0.9 mmol) in dichloromethane (30 mL) at 273 K. After being stirred at room temperature for 4h, the mixture was washed with saturated sodium bicarbonate solution and the organic layer was separated, dried over magnesium sulfate, filtered and concentrated at reduced pressure. The residue was purified by column chromatography (hexane–ethyl acetate, 2:1 v/v) to afford the title compound as a colorless solid [yield 76%, m.p. 464–465 K; *R*_f_ = 0.54 (hexane–ethyl acetate, 2:1 v/v)]. Single crystals suitable for X-ray diffraction were prepared by slow evaporation of a solution of the title compound in acetone at room temperature.

### Refinement

All H atoms were positioned geometrically and refined using a riding model, with C—H = 0.95 Å for aryl and methine, 0.99 Å for methylene and 0.98 Å for methyl H atoms, respectively. *U*_iso_(H) = 1.2*U*_eq_(C) for aryl, methine and methylene, and 1.5*U*_eq_(C) for methyl H atoms.

### Crystal data

**Table:**

C_21_H_21_FO_2_S	*F*(000) = 1504
*M**_r_* = 356.44	*D*_x_ = 1.353 Mg m^−^^3^
Orthorhombic, *P**b**c**a*	Mo *K*α radiation, λ = 0.71073 Å
Hall symbol: -P 2ac 2ab	Cell parameters from 4239 reflections
*a* = 16.4070 (6) Å	θ = 2.2–27.3°
*b* = 11.3751 (4) Å	µ = 0.21 mm^−^^1^
*c* = 18.7490 (7) Å	*T* = 173 K
*V* = 3499.1 (2) Å^3^	Block, colourless
*Z* = 8	0.26 × 0.17 × 0.14 mm

### Data collection

**Table:**

Bruker SMART APEXII CCD diffractometer	4007 independent reflections
Radiation source: rotating anode	3190 reflections with *I* > 2σ(*I*)
graphite multilayer	*R*_int_ = 0.041
Detector resolution: 10.0 pixels mm^-1^	θ_max_ = 27.5°, θ_min_ = 2.2°
φ and ω scans	*h* = −21→16
Absorption correction: multi-scan (*SADABS*; Bruker, 2009)	*k* = −14→14
*T*_min_ = 0.949, *T*_max_ = 0.972	*l* = −24→23
17170 measured reflections	

### Refinement

**Table:**

Refinement on *F*^2^	Primary atom site location: structure-invariant direct methods
Least-squares matrix: full	Secondary atom site location: difference Fourier map
*R*[*F*^2^ > 2σ(*F*^2^)] = 0.040	Hydrogen site location: difference Fourier map
*wR*(*F*^2^) = 0.112	H-atom parameters constrained
*S* = 0.93	*w* = 1/[σ^2^(*F*_o_^2^) + (0.0621*P*)^2^ + 1.9145*P*] where *P* = (*F*_o_^2^ + 2*F*_c_^2^)/3
4007 reflections	(Δ/σ)_max_ < 0.001
227 parameters	Δρ_max_ = 0.32 e Å^−^^3^
0 restraints	Δρ_min_ = −0.44 e Å^−^^3^

### Special details

**Table:**

Geometry. All esds (except the esd in the dihedral angle between two l.s. planes) are estimated using the full covariance matrix. The cell esds are taken into account individually in the estimation of esds in distances, angles and torsion angles; correlations between esds in cell parameters are only used when they are defined by crystal symmetry. An approximate (isotropic) treatment of cell esds is used for estimating esds involving l.s. planes.
Refinement. Refinement of F^2^ against ALL reflections. The weighted R-factor wR and goodness of fit S are based on F^2^, conventional R-factors R are based on F, with F set to zero for negative F^2^. The threshold expression of F^2^ > 2sigma(F^2^) is used only for calculating R-factors(gt) etc. and is not relevant to the choice of reflections for refinement. R-factors based on F^2^ are statistically about twice as large as those based on F, and R- factors based on ALL data will be even larger.

### Fractional atomic coordinates and isotropic or equivalent isotropic displacement parameters (Å^2^)

**Table:**

	*x*	*y*	*z*	*U*_iso_*/*U*_eq_	
S1	0.22808 (2)	0.03255 (4)	0.46770 (2)	0.02460 (12)	
F1	0.19074 (7)	−0.29503 (10)	0.76480 (6)	0.0432 (3)	
O1	0.42443 (7)	0.03716 (10)	0.59102 (6)	0.0241 (3)	
O2	0.20712 (7)	0.14989 (11)	0.43665 (7)	0.0324 (3)	
C1	0.32425 (9)	0.04757 (14)	0.50865 (8)	0.0215 (3)	
C2	0.38848 (9)	0.12710 (14)	0.48685 (8)	0.0215 (3)	
C3	0.39949 (9)	0.20728 (14)	0.43111 (8)	0.0220 (3)	
H3	0.3591	0.2159	0.3952	0.026*	
C4	0.47040 (9)	0.27410 (15)	0.42914 (8)	0.0231 (3)	
C5	0.52971 (9)	0.25870 (15)	0.48281 (8)	0.0256 (3)	
H5	0.5784	0.3037	0.4805	0.031*	
C6	0.51959 (10)	0.18048 (16)	0.53860 (8)	0.0259 (4)	
H6	0.5598	0.1711	0.5746	0.031*	
C7	0.44797 (9)	0.11676 (15)	0.53917 (8)	0.0227 (3)	
C8	0.34835 (10)	−0.00304 (15)	0.57127 (8)	0.0227 (3)	
C9	0.30871 (10)	−0.08261 (14)	0.62133 (8)	0.0231 (3)	
C10	0.32514 (10)	−0.07323 (15)	0.69434 (8)	0.0246 (3)	
H10	0.3633	−0.0165	0.7106	0.030*	
C11	0.28651 (10)	−0.14560 (15)	0.74306 (8)	0.0272 (4)	
H11	0.2980	−0.1402	0.7926	0.033*	
C12	0.23105 (11)	−0.22544 (15)	0.71749 (9)	0.0286 (4)	
C13	0.21323 (11)	−0.23782 (16)	0.64647 (9)	0.0310 (4)	
H13	0.1742	−0.2940	0.6310	0.037*	
C14	0.25320 (11)	−0.16691 (15)	0.59778 (8)	0.0292 (4)	
H14	0.2428	−0.1757	0.5482	0.035*	
C15	0.48353 (9)	0.35926 (14)	0.36836 (8)	0.0229 (3)	
H15	0.4301	0.3682	0.3433	0.028*	
C16	0.51036 (12)	0.48237 (16)	0.39185 (9)	0.0314 (4)	
H16A	0.4695	0.5153	0.4252	0.038*	
H16B	0.5631	0.4770	0.4173	0.038*	
C17	0.51939 (12)	0.56396 (17)	0.32774 (10)	0.0364 (4)	
H17A	0.4654	0.5758	0.3053	0.044*	
H17B	0.5395	0.6415	0.3440	0.044*	
C18	0.57831 (12)	0.51361 (19)	0.27288 (11)	0.0406 (5)	
H18A	0.6338	0.5108	0.2936	0.049*	
H18B	0.5799	0.5658	0.2306	0.049*	
C19	0.55313 (12)	0.39042 (18)	0.24985 (9)	0.0370 (4)	
H19A	0.5007	0.3944	0.2238	0.044*	
H19B	0.5947	0.3579	0.2171	0.044*	
C20	0.54405 (11)	0.30958 (16)	0.31408 (9)	0.0293 (4)	
H20A	0.5978	0.2992	0.3372	0.035*	
H20B	0.5250	0.2314	0.2979	0.035*	
C21	0.26034 (12)	−0.05707 (17)	0.39440 (9)	0.0338 (4)	
H21A	0.2153	−0.0659	0.3605	0.051*	
H21B	0.2766	−0.1347	0.4121	0.051*	
H21C	0.3067	−0.0197	0.3705	0.051*	

### Atomic displacement parameters (Å^2^)

**Table:**

	*U*^11^	*U*^22^	*U*^33^	*U*^12^	*U*^13^	*U*^23^
S1	0.0214 (2)	0.0258 (2)	0.0266 (2)	−0.00355 (16)	−0.00049 (14)	0.00255 (16)
F1	0.0587 (7)	0.0383 (7)	0.0326 (6)	−0.0129 (6)	0.0100 (5)	0.0092 (5)
O1	0.0261 (6)	0.0242 (6)	0.0221 (5)	−0.0022 (5)	−0.0018 (4)	0.0027 (4)
O2	0.0283 (6)	0.0280 (7)	0.0411 (7)	0.0044 (5)	−0.0038 (5)	0.0052 (6)
C1	0.0241 (7)	0.0195 (8)	0.0208 (7)	−0.0006 (6)	0.0002 (6)	−0.0027 (6)
C2	0.0212 (7)	0.0208 (8)	0.0224 (7)	−0.0001 (6)	0.0002 (5)	−0.0035 (6)
C3	0.0219 (7)	0.0239 (8)	0.0201 (7)	−0.0008 (6)	−0.0009 (5)	−0.0011 (6)
C4	0.0238 (7)	0.0227 (9)	0.0228 (7)	0.0008 (6)	0.0020 (6)	−0.0006 (6)
C5	0.0217 (7)	0.0276 (9)	0.0273 (8)	−0.0034 (7)	−0.0006 (6)	−0.0013 (7)
C6	0.0233 (7)	0.0297 (9)	0.0248 (8)	−0.0004 (7)	−0.0039 (6)	−0.0010 (7)
C7	0.0253 (7)	0.0221 (8)	0.0207 (7)	0.0020 (7)	0.0005 (6)	−0.0002 (6)
C8	0.0248 (7)	0.0203 (8)	0.0230 (7)	−0.0005 (6)	−0.0006 (6)	−0.0019 (6)
C9	0.0277 (8)	0.0183 (8)	0.0232 (7)	0.0011 (6)	0.0011 (6)	0.0007 (6)
C10	0.0280 (8)	0.0222 (9)	0.0237 (7)	0.0009 (7)	−0.0020 (6)	−0.0004 (6)
C11	0.0343 (9)	0.0264 (9)	0.0209 (7)	0.0055 (7)	0.0007 (6)	0.0013 (7)
C12	0.0374 (9)	0.0218 (9)	0.0265 (8)	0.0018 (7)	0.0082 (6)	0.0049 (7)
C13	0.0408 (9)	0.0224 (9)	0.0299 (9)	−0.0071 (8)	−0.0003 (7)	−0.0013 (7)
C14	0.0415 (9)	0.0244 (9)	0.0218 (7)	−0.0028 (8)	−0.0013 (7)	−0.0008 (7)
C15	0.0224 (7)	0.0226 (8)	0.0238 (7)	−0.0018 (6)	0.0006 (6)	0.0018 (6)
C16	0.0389 (9)	0.0244 (9)	0.0309 (9)	−0.0017 (8)	0.0008 (7)	−0.0033 (7)
C17	0.0420 (10)	0.0249 (10)	0.0423 (10)	−0.0038 (8)	−0.0001 (8)	0.0046 (8)
C18	0.0394 (10)	0.0375 (11)	0.0449 (11)	−0.0041 (9)	0.0089 (8)	0.0146 (9)
C19	0.0437 (10)	0.0384 (11)	0.0291 (8)	0.0045 (9)	0.0095 (7)	0.0057 (8)
C20	0.0340 (9)	0.0264 (9)	0.0277 (8)	0.0014 (7)	0.0050 (6)	−0.0009 (7)
C21	0.0443 (10)	0.0270 (9)	0.0302 (8)	−0.0018 (8)	−0.0083 (7)	−0.0053 (7)

### Geometric parameters (Å, °)

**Table:**

S1—O2	1.4959 (13)	C12—C13	1.371 (2)
S1—C1	1.7633 (16)	C13—C14	1.384 (2)
S1—C21	1.7910 (18)	C13—H13	0.9500
F1—C12	1.3607 (18)	C14—H14	0.9500
O1—C8	1.3800 (19)	C15—C20	1.530 (2)
O1—C7	1.3836 (19)	C15—C16	1.533 (2)
C1—C8	1.366 (2)	C15—H15	1.0000
C1—C2	1.448 (2)	C16—C17	1.526 (2)
C2—C7	1.389 (2)	C16—H16A	0.9900
C2—C3	1.399 (2)	C16—H16B	0.9900
C3—C4	1.390 (2)	C17—C18	1.523 (3)
C3—H3	0.9500	C17—H17A	0.9900
C4—C5	1.411 (2)	C17—H17B	0.9900
C4—C15	1.511 (2)	C18—C19	1.523 (3)
C5—C6	1.383 (2)	C18—H18A	0.9900
C5—H5	0.9500	C18—H18B	0.9900
C6—C7	1.381 (2)	C19—C20	1.522 (2)
C6—H6	0.9500	C19—H19A	0.9900
C8—C9	1.457 (2)	C19—H19B	0.9900
C9—C14	1.394 (2)	C20—H20A	0.9900
C9—C10	1.399 (2)	C20—H20B	0.9900
C10—C11	1.383 (2)	C21—H21A	0.9800
C10—H10	0.9500	C21—H21B	0.9800
C11—C12	1.372 (2)	C21—H21C	0.9800
C11—H11	0.9500		
			
O2—S1—C1	106.78 (7)	C13—C14—H14	119.9
O2—S1—C21	106.10 (8)	C9—C14—H14	119.9
C1—S1—C21	97.17 (8)	C4—C15—C20	110.95 (13)
C8—O1—C7	106.31 (11)	C4—C15—C16	114.25 (13)
C8—C1—C2	107.21 (13)	C20—C15—C16	110.01 (14)
C8—C1—S1	126.32 (12)	C4—C15—H15	107.1
C2—C1—S1	126.07 (12)	C20—C15—H15	107.1
C7—C2—C3	119.46 (14)	C16—C15—H15	107.1
C7—C2—C1	105.02 (14)	C17—C16—C15	110.99 (14)
C3—C2—C1	135.46 (14)	C17—C16—H16A	109.4
C4—C3—C2	119.00 (14)	C15—C16—H16A	109.4
C4—C3—H3	120.5	C17—C16—H16B	109.4
C2—C3—H3	120.5	C15—C16—H16B	109.4
C3—C4—C5	119.36 (15)	H16A—C16—H16B	108.0
C3—C4—C15	119.36 (13)	C18—C17—C16	111.38 (15)
C5—C4—C15	121.26 (14)	C18—C17—H17A	109.4
C6—C5—C4	122.44 (15)	C16—C17—H17A	109.4
C6—C5—H5	118.8	C18—C17—H17B	109.4
C4—C5—H5	118.8	C16—C17—H17B	109.4
C7—C6—C5	116.47 (14)	H17A—C17—H17B	108.0
C7—C6—H6	121.8	C17—C18—C19	111.44 (15)
C5—C6—H6	121.8	C17—C18—H18A	109.3
C6—C7—O1	125.93 (14)	C19—C18—H18A	109.3
C6—C7—C2	123.25 (15)	C17—C18—H18B	109.3
O1—C7—C2	110.81 (13)	C19—C18—H18B	109.3
C1—C8—O1	110.65 (13)	H18A—C18—H18B	108.0
C1—C8—C9	133.35 (14)	C20—C19—C18	110.97 (15)
O1—C8—C9	115.89 (13)	C20—C19—H19A	109.4
C14—C9—C10	119.18 (15)	C18—C19—H19A	109.4
C14—C9—C8	121.00 (14)	C20—C19—H19B	109.4
C10—C9—C8	119.81 (14)	C18—C19—H19B	109.4
C11—C10—C9	120.83 (15)	H19A—C19—H19B	108.0
C11—C10—H10	119.6	C19—C20—C15	111.52 (14)
C9—C10—H10	119.6	C19—C20—H20A	109.3
C12—C11—C10	117.84 (14)	C15—C20—H20A	109.3
C12—C11—H11	121.1	C19—C20—H20B	109.3
C10—C11—H11	121.1	C15—C20—H20B	109.3
F1—C12—C13	118.01 (16)	H20A—C20—H20B	108.0
F1—C12—C11	118.67 (14)	S1—C21—H21A	109.5
C13—C12—C11	123.31 (15)	S1—C21—H21B	109.5
C12—C13—C14	118.66 (16)	H21A—C21—H21B	109.5
C12—C13—H13	120.7	S1—C21—H21C	109.5
C14—C13—H13	120.7	H21A—C21—H21C	109.5
C13—C14—C9	120.15 (15)	H21B—C21—H21C	109.5
			
O2—S1—C1—C8	141.75 (14)	C7—O1—C8—C9	−175.83 (13)
C21—S1—C1—C8	−108.99 (15)	C1—C8—C9—C14	32.7 (3)
O2—S1—C1—C2	−30.00 (15)	O1—C8—C9—C14	−151.68 (15)
C21—S1—C1—C2	79.26 (15)	C1—C8—C9—C10	−146.03 (18)
C8—C1—C2—C7	0.58 (17)	O1—C8—C9—C10	29.6 (2)
S1—C1—C2—C7	173.63 (12)	C14—C9—C10—C11	−0.6 (2)
C8—C1—C2—C3	−176.33 (17)	C8—C9—C10—C11	178.17 (15)
S1—C1—C2—C3	−3.3 (3)	C9—C10—C11—C12	−0.8 (2)
C7—C2—C3—C4	0.6 (2)	C10—C11—C12—F1	−178.13 (15)
C1—C2—C3—C4	177.17 (17)	C10—C11—C12—C13	1.0 (3)
C2—C3—C4—C5	0.6 (2)	F1—C12—C13—C14	179.42 (16)
C2—C3—C4—C15	178.85 (14)	C11—C12—C13—C14	0.3 (3)
C3—C4—C5—C6	−1.1 (2)	C12—C13—C14—C9	−1.8 (3)
C15—C4—C5—C6	−179.33 (15)	C10—C9—C14—C13	1.9 (3)
C4—C5—C6—C7	0.4 (2)	C8—C9—C14—C13	−176.85 (16)
C5—C6—C7—O1	−177.69 (15)	C3—C4—C15—C20	−103.02 (17)
C5—C6—C7—C2	0.9 (2)	C5—C4—C15—C20	75.18 (19)
C8—O1—C7—C6	178.41 (16)	C3—C4—C15—C16	131.93 (16)
C8—O1—C7—C2	−0.35 (17)	C5—C4—C15—C16	−49.9 (2)
C3—C2—C7—C6	−1.4 (2)	C4—C15—C16—C17	−178.01 (14)
C1—C2—C7—C6	−178.94 (15)	C20—C15—C16—C17	56.44 (19)
C3—C2—C7—O1	177.37 (13)	C15—C16—C17—C18	−56.0 (2)
C1—C2—C7—O1	−0.14 (17)	C16—C17—C18—C19	55.1 (2)
C2—C1—C8—O1	−0.83 (18)	C17—C18—C19—C20	−54.9 (2)
S1—C1—C8—O1	−173.86 (11)	C18—C19—C20—C15	56.2 (2)
C2—C1—C8—C9	174.93 (17)	C4—C15—C20—C19	175.80 (14)
S1—C1—C8—C9	1.9 (3)	C16—C15—C20—C19	−56.79 (18)
C7—O1—C8—C1	0.74 (17)		

### Hydrogen-bond geometry (Å, °)

**Table:**

Cg1 and Cg2 are the centroids of the C1/C2/C7/O1/C8 furan ring and C9–C14 4-fluorophenyl ring.

**Table:**

*D*—H···*A*	*D*—H	H···*A*	*D*···*A*	*D*—H···*A*
C21—H21B···O2^i^	0.98	2.51	3.468 (2)	166
C13—H13···Cg1^i^	0.95	2.55	3.427 (2)	153
C20—H20A···Cg2^ii^	0.99	2.73	3.537 (2)	140

Symmetry codes: (i) −*x*+1/2, *y*−1/2, *z*; (ii) −*x*+1, −*y*, −*z*+1.

## References

[bb1] Akgul, Y. Y. & Anil, H. (2003). *Phytochemistry*, **63**, 939-943. 10.1016/s0031-9422(03)00357-1 12895543

[bb2] Aslam, S. N., Stevenson, P. C., Phythian, S. J., Veitch, N. C. & Hall, D. R. (2006). *Tetrahedron*, **62**, 4214–4226.

[bb3] Brandenburg, K. (1998). *DIAMOND* Crystal Impact GbR, Bonn, Germany.

[bb4] Bruker (2009). *APEX2*, *SADABS* and *SAINT* Bruker AXS Inc., Madison, Wisconsin, USA.

[bb5] Choi, H. D., Seo, P. J., Son, B. W. & Lee, U. (2009). *Acta Cryst.* E**65**, o2649. 10.1107/S1600536809039749 PMC2971000 21578263

[bb6] Choi, H. D., Seo, P. J., Son, B. W. & Lee, U. (2010*a*). *Acta Cryst.* E**66**, o44. 10.1107/S1600536809051642 PMC2980179 21580148

[bb7] Choi, H. D., Seo, P. J., Son, B. W. & Lee, U. (2010*b*). *Acta Cryst.* E**66**, o104. 10.1107/S1600536809052519 PMC2980067 21579995

[bb8] Farrugia, L. J. (1997). *J. Appl. Cryst.* **30**, 565.

[bb9] Galal, S. A., Abd El-All, A. S., Abdallah, M. M. & El-Diwani, H. I. (2009). *Bioorg. Med. Chem. Lett* **19**, 2420–2428. 10.1016/j.bmcl.2009.03.069 19345581

[bb10] Khan, M. W., Alam, M. J., Rashid, M. A. & Chowdhury, R. (2005). *Bioorg. Med. Chem* **13**, 4796–4805. 10.1016/j.bmc.2005.05.009 15964760

[bb11] Sheldrick, G. M. (2008). *Acta Cryst.* A**64**, 112–122. 10.1107/S0108767307043930 18156677

[bb12] Soekamto, N. H., Achmad, S. A., Ghisalberti, E. L., Hakim, E. H. & Syah, Y. M. (2003). *Phytochemistry*, **64**, 831–834. 10.1016/j.phytochem.2003.08.009 14559276

